# Corneal cross linking and infectious keratitis: a systematic review with a meta-analysis of reported cases

**DOI:** 10.1186/1869-5760-3-47

**Published:** 2013-05-29

**Authors:** Jorge L Alio, Alessandro Abbouda, David Diaz Valle, Jose M Benitez del Castillo, Jose A Gegundez Fernandez

**Affiliations:** 1 Vissum Corporación Oftalmológica, Alicante 03016, Spain; 2 Division of Ophthalmology, Universidad Miguel Hernández, Alicante 03016, Spain; 3 Unidad de Superficie e Inflamación Ocular, Servicio de Oftalmología, Hospital Clínico San Carlos, Universidad Complutense de Madrid, Madrid 28040, Spain

**Keywords:** Collagen cross linking, Infectious keratitis, UV-A, Corneal melting

## Abstract

**Background:**

Collagen cross linking (CXL) of the cornea has been developed recently as a new treatment for multidrug-resistant infectious keratitis. The aim of this study is to summarize the previously published data and evaluate the effectiveness of this treatment.

**Results:**

The search identified 12 articles. The number of eyes was 104. The infectious keratitis was associated with bacteria in 58 eyes (57f%): Gram-positive bacteria in 44 (43%; 4 of which were infected with *Mycobacterium* (3.6%)) and Gram-negative bacteria in 14 eyes (13%), fungus in 13 eyes (12%), and *Acanthamoeba* in 7 eyes (7%). In 26 eyes (25%), the microbiological culture was negative or not performed. The mean time of re-epithelization after CXL was 20.7±28.1 days (minimum of 3, maximum of 145). Sixteen eyes underwent deep or lamellar keratoplasty. The pooled analysis suggested that CXL has a favorable effect on the block of corneal melting in 85% (95%; CI 0.77, 0.91) of eyes.

**Conclusion:**

Although randomized controlled trials are needed, the available evidence supports the use of CXL in the treatment of infectious keratitis.

## Background

Microbial keratitis is an infection of the cornea that is associated with a risk of permanent visual impairment [1-5]. It can be caused by bacteria, virus, fungus, protozoa and parasites. The common risk factors for infectious keratitis include ocular trauma, contact lens wear, recent ocular surgery, preexisting ocular surface disease, dry eyes, lid deformity, corneal sensation impairment, chronic use of topical steroids and systemic immunosuppression [6-8]. The treatment usually consists of topically administered antibiotics. If diagnosis and initiation of appropriate antimicrobial treatment are delayed, it has been estimated that only 50% of the eyes will heal with good visual outcome [9]. The emergence of multidrug-resistant bacteria is a concern that might complicate the treatment and cure of infectious keratitis considerably. Microbes develop resistance to antibiotics as a result of chromosomal mutation, inductive expression of latent chromosomal genes, or exchange of genetic material via transformation, bacteriophage transduction, or plasmid conjugation [10-12]. Some microbial keratitis resistant to the newest antibiotics have been recently described [12,13]. The high cost of this drug, high frequency of resistance to antibiotics, and risk of corneal melting and corneal scars make the choice of new approaches very desirable. Collagen cross linking (CXL) using ultraviolet-A (UV-A) and riboflavin is a treatment that was developed to increase the biomechanical strength of the cornea thus giving it possibilities to block the progression of keratoconus [14,15]. The procedure is based on using riboflavin as a photosensitizer, which generates reactive oxygen species when activated by UV-A at 365 or 370 nm. By way of photochemical reactions, these give rise to covalent bonds or cross-links in the corneal stroma. Riboflavin, or vitamin B2, is a naturally occurring compound and an essential human nutrient. Japanese scientists demonstrated in the 1960s that riboflavin, when exposed to visible or UV light, could be used to inactivate the RNA containing tobacco mosaic virus [16]. Research has been underway since 2000 in using riboflavin as a photosensitizer to inactivate pathogens in plasma, platelet and red cell products [17-19]. The photoactivation of riboflavin damages the RNA and DNA of microorganisms by oxidation processes, causing lesions in chromosomal strands [17-20]. Riboflavin has a planar ring structure that intercalates between bases of DNA and RNA, which results in the oxidation of nucleic acids when illuminated. Riboflavin induces a change in properties of the collagen and has a stiffening effect on the corneal stroma, which stabilizes it and increases its resistance to enzymatic bacteria degradation avoiding the progression of corneal melting [21,22]. Limitations of the application of UV are mainly the lack of penetration and a strong dependence on the distance from the UV source, which may result in nonhomogeneous microbial inactivation. The complications related to UV light should be carefully considered in patients with corneal ulcers. Usually, only <10% of UV light penetrates the anterior chamber and interacts with the aqueous that contains a small concentration of riboflavin. In these patients, the penetration would be greater and would cause endothelial cell loss. A number of publications [23-29] have described that CXL was able to improve healing in patients with microbial keratitis and to block the progression of corneal melting.

*In vitro* experiments [30-32] have supported the view that there is a bactericidal effect of this combination by demonstration of bacterial elimination using a 365-nm light photo activation of riboflavin. We analyze the studies published on severe infectious keratitis treated with the corneal cross linking procedure. The results indicate that this could be a new tool in the management of infectious keratitis resistant to antibiotic treatment. In 2000, Schnitzler [33] first described the use of CXL to four patients suffering from melting ulcer of the cornea of various origins. After the treatment of three of the four patients, the melting process was blocked; thereafter, several cases [23-25,27-29,34-38] are described, but a systematic analysis of these data is missing.

## Methods

Published journal articles were considered as the elements of study and a specific literature search was performed in four stages:

Stage 1 (Unique citations) - A *Medline* (National Library of Medicine, Bethesda, Maryland, USA) search from January 2000 to January 2013 was performed to identify all articles describing the treatment of CXL in infectious keratitis. Keyword searches used were the terms ‘cross linking’?+?‘keratitis’ limit to ‘2000 to 2013’, and [‘Ulcerative’ or ‘Microbial’]?+?‘keratitis’?+?‘cross linking’ limit to ‘2000 to 2013’.

Stage 2 (Article retrieval) - All abstracts from the *Medline* searches were scrutinized to identify articles that reported clinical results. We excluded *in vitro* or animal studies. Only journal articles published in English were included. Copies of the articles were obtained, and their bibliographies were searched manually for additional articles published in peer-reviewed journals.

Stage 3 (Article inclusion) - Complete articles were reviewed to identify those that reported original clinical data or complication(s) of CXL treatment. As the numbers of articles were so few, we decided to include all.

Stage 4 (Article exclusion) - We excluded all articles that described the use of CXL *in vitro* or in animals.

### Data abstractions and analysis

A meticulous and systematic review of the complete articles was performed. All appropriate information regarding aspects of CXL treatment was analyzed. The primary outcome was the healing of corneal ulcer, defined as epithelization, with no progressive infiltration, block of corneal melting and secondary end-point recovery of visual acuity. We evaluated the total, partial and incomplete resolutions of corneal ulcer after the procedure. Adverse outcomes are the progression of melting, corneal transplants, corneal decompensation and complications related to the procedure. All data were analyzed using Microsoft Excel (Microsoft Corporation, Washington, USA). Patient population characteristics were recorded. Complications and their treatment were noted. To determine the incidence of block corneal melting after CXL, the sum of block corneal melting was divided by the total number of patients for each study. There were 12 articles on CXL treatment in infectious keratitis [23-29,34-38]. According to the protocol for the Cochrane systematic meta-analysis [39], all the articles included in this study were classified as level 3 evidence (non-analytical studies: case reports, case series). To assess the validity of each study, we evaluated the similarity of groups at the baseline, the description of primary and secondary outcomes and the presentation of the results. Furthermore, the adequacy of reporting data was evaluated and the missing follow up was taken into account. These parameters were classified as adequate, partial inadequate and unknown. The risk of bias was evaluated. When all the criteria were adequate, the study was classified as having a low risk of bias. Within this group, we found three studies [24,34,37]. When one or more criteria were partially met, we classified the study as having a moderate risk of bias. Four studies were included in this category [25,27,29,36]. In five studies [23,26,28,35,38], one or more criteria did not meet the guideline and these studies were classified as having a high risk of bias.

## Results and discussion

### Results

A total of 12 articles were selected. Table 1 summarizes data items from each paper. All papers were case reports or case series. The total number of eyes treated with CXL was 104. As none of the studies was analytical, comparative statistical methods could not be applied. Price, in his article [34], suggests that 200 participants would be required to adequately test the hypothesis of the effect of CXL on infectious keratitis and the enrolment of the patients in this study is still in progress. A UV-X lamp (PESCHKE Meditrade GmbH, Hueneberg, Switzerland) was the instrument used in most cases. Nine studies [23-27,34,36-38] performed the procedure using this instrument. A Vega CBM X linker (CSO, Florence, Italy) was used in one study [28], and in two studies [29,35], the models of the instruments were not specified. Standard treatment and parameters were used in all cases. A 365-nm wavelength of UV-A light source was used to irradiate the ulcer, carefully avoiding the limbal area. Corneal surface irradiance was approximately 3 mW/cm^2^ for a period of 30min. In all cases [23-25,27-29,34-38], during the induction period, 0.1% riboflavin and 20% dextran T500 drops were topically administered to the cornea for a period of 20 to 30min at intervals of 2 to 3min. During the irradiance, the administration of riboflavin was continued with one drop at 5-min intervals. In one study [37], hypotonic riboflavin was used when corneal thickness was less than 400μm, and only in one article [34], 36 patients were randomized to different UV-A durations using a web-based random number generator. The first 12 participants were randomized to 15 or 30min. The protocol was amended, and subsequent participants (*n*?=?24) were randomized to 30- or 45-min treatments after Theo Seiler, M.D., Ph.D., suggested that duration should perhaps be lengthened for infectious keratitis (personal communication, April 12, 2010). Forty one subjects were male and 44 female; in some cases, the gender was not specified. The average age was 52.8?±?19. It was impossible to extract detailed data of visual acuity from some studies. At the first visit, the average best-corrected visual acuity (BCVA) was 0.03?±?0.05 and at the end of follow up was 0.16?±?0.17. The best results of BCVA are obtained in one patient infected with *Moraxella* and two with *S. aureus*. The worst were in patients infected with a fungus and *Mycobacterium*. The infectious keratitis was associated with bacteria in 58 eyes (57%): Gram-positive bacteria in 44 (43%; 4 of which were infected with *Mycobacterium* (3.6%)) and Gram-negative bacteria in 14 eyes (13%), fungus in 13 eyes (12%), and *Acanthamoeba* in 7 eyes (7%). In 26 eyes (25%), the microbiological culture was negative or not performed. The size of the infiltrate ranged between 0.1 and 8.5mm with an average of 2.7mm. At the first visit, 16 eyes presented hypopyon. The mean time of re-epithelization after CXL was 20.7?±?28.1days (minimum of 3; maximum of 145). It is possible to detect a long period of epithelization in older patients (Pearson −0.1, *p*?<?0.4) and in patients infected with *Acanthamoeba* and *Aspergillus* (60 to 140days) and *Mycobacterium* (120days). We observed faster epithelization in Gram-positive bacteria followed by Gram-negative bacteria, *Acanthamoeba* and fungus. The duration of epithelization divided by each group of pathogens are summarized in Table 2. We observed very few complications after the CXL procedure. One eye developed corneal edema and one had a severe dendritic lesion. The first eye was affected with *Candida* and the second had a negative culture. The antibiotic and the antifungal treatment were continued after CXL for 20?±?26.9days. The patients (16 eyes) from the Makdoumi [24] study did not have antibiotic drops. *Acanthamoeba* treatment was followed for 50days after CXL. Sixteen eyes underwent deep or lamellar keratoplasty. Corneal transplantation was more frequent in fungus followed by *Acanthamoeba* and Gram-positive and Gram-negative bacteria. Table 3 describes the incidence of transplantation in each group. Eleven patients needed corneal transplantation for tectonic reasons; the other five patients only needed visual rehabilitation due to residual cornea scars. We estimated that the overall probability of blocking corneal melting and promoting epithelization in patients affected by infectious keratitis treated with CXL was 85% (95%; CI 0.77, 0.91). The estimate of response was quite similar between the studies (Table 4).

**Table 1:** Summary of published study

**Authors**	**Makdoumi [**[25]**]**	**Makdoumi [**[24]**]**	**Morén [**[26]**]**	**Iseli [**[27]**]**	**Micelli Ferrari [**[28]**]**	**Khan [**[29]**]**	**Anwar [**[23]**]**	**Price [**[34]**]**	**Panda [**[35]**]**	**Sorkhabi [**[36]**]**	**Müller [**[37]**]**	**Skaat [**[38]**]**
Years published	2010	2011	2010	2008	2009	2011	2011	2012	2012	2013	2012	2011
Design	Case series	Case series	Case report	Case series	Case report	Case series	Case series	Case series	Case series	Case Series	Case series	Case series
Level of evidence	3	3	3	3	3	3	3	3	3	3	3	3
Risk of bias	B	A	C	B	C	B	C	A	C	B	A	C
Number of patients	7	16	1	5	1	3	2	40	7	10	6	6
Age	66.7 (43 to 84)	42 (18 to 83)	25	43.2 (27 to 66)	78	42 (30 to 61)	38.5 (30 to 47)	57.1 (18 to 86)	50 (18 to 72)	62.4 (18 to 78)	41.8 (16 to 78)	46.3 (21 to 66)
Bacteria	4	12	0	3	1	0	1	24	0	9	1	3
*Gram*-*positive*	*2*	*12*		*3*			*1*	*15*		*9*		*2*
*Gram*-*negative*	*2*				*1*			*9*			*1*	*1*
Fungus	0	0	0	2	0	0	1	7	0	1	1	0
*Yeas*t											*1*	
*Mold*				*2*			*1*	*7*		*1*		
Protozoan	0	0	0	0	0	3	0	2	1	0	1	0
*Acanthamoeba*						*3*		*2*	*1*		*1*	
Virus	0	0	0	0	0	0	0	1	0	0	0	0
Negative/not performed	3	4	1	0	0	0	0	6	6	0	3	3
Melting arrested	7	15	1	4	1	3	2	34	7	8	4	5
Cornea transplanted	1	0	0	3	0	2	1	5	0	1	2	1
*Visual rehabilitation*				2						(*1 enucleation*)		
*Tectonic aim*	1			1		2	1	5		1	2	1

Level 3 evidence?=?non-analytical studies; Risk of bias: A, low risk of bias; B, moderate risk of bias; C, high risk of bias.

**Table 2:** Time of epithelization in days among different infections after CXL

**Pathogens**	**Time in days**
Gram-positive bacteria	11.1 (3 to 45)
Gram-negative bacteria	12.6 (4 to 30)
Fungus	53.2 (3 to 120)
*Acanthamoeba*	66.6 (8 to 145)
Negative	19.25 (4 to 90)

**Table 3:** Cornea transplant related to different pathogens

**Microbial pathogens**	**Number of eyes transplanted among eyes infected with the same pathogen (%, IC 95%)**	
Gram-positive bacteria	7/44 16%	(0.06 to 0.27)
Gram-negative bacteria	1/14 7%	(0.01 to 0.33)
*Acanthamoeba*	2/7 28%	(0.08 to 0.64)
Mold	4/12 33%	(0.13 to 0.6)
Yeast	1/1 100%	(0.2 to 1)
Negative	1/26 4%	(0.008 to 0.2)
Total	16/104 15%	(0.09 to 0.23)

**Table 4:** Proportion of eye responding to CXL in each study

**Reference/country**	**Proportion of eyes improved with CXL**	**IC 95%**
Makdoumi et al. (2010)/Sweden [25]	6/7	0.85 (0.48 to 0.97)
Makdoumi et al. (2011)/Sweden [24]	15/16	0.93 (0.7 to 0.98)
Morén (2008)/Sweden [26]	1/1	1 (0.2 to 1)
Iseli et al. (2008)/Switzerland [27]	4/5	0.8 (0.37 to 0.96)
Micelli Ferrari et al. (2009)/Italy [28]	1/1	1 (0.2 to 1)
Khan et al. (2011)/Maryland [29]	3/3	1 (0.43 to 1)
Anwar et al. (2011)/Saudi Arabia [23]	1/2	0.5 (0.09 to 0.9)
Price et al. (2012)/USA [34]	34/40	0.85 (07 to 0.92)
Panda et al. (2012)/India [35]	7/7	1 (0.6 to 1)
Sorkhabi et al. (2013)/Iran [36]	8/10	0.8 (0.49 to 0.94)
Müller et al. (2012)/Switzerland [37]	4/6	0.6 (0.3 to 0.9)
Skaat et al. (2011)/Israel [38]	5/6	0.8 (0.43 to 0.96)
Total studies	89/104	0.85 (0.77 to 0.91)

### Discussion

Riboflavin and its degradation products have been studied for a long time and have very high safety profiles [7,15]. With the current applications and properties of the UV-A and B2 therapy in mind, we ventured to explore its potential use for treating infectious keratitis. Martins et al. [22] reported *in vitro* a wide spectrum of microorganism eradication. There seems to be strong evidence that the process could also be effective in infectious keratitis. According to previous studies [23-25,27-29,34-38], the treatment seems more effective to block corneal melting in the following order: in Gram-negative bacteria (13/14; 92%) followed by Gram-positive bacteria (37/44; 84%), *Acanthamoeba* (5/7; 71%), and finally fungus (8/13; 61%). In the Gram-positive groups, the bacterium with the worst outcome was *Mycobacterium*. All patients infected with this underwent corneal transplants. In *Acanthamoeba*, the free radicals generated by irradiating B2, combined with hydrogen peroxide-based oxidation, seem to be successful in neutralizing Acanthamoeba cyst [29]. At the moment, the indication of this treatment is only for infectious keratitis not responsive to antibiotic treatment, but we can start to think about using it as an alternative treatment, especially for *Acanthamoeba*. The most important exclusion criterion is the depth of infiltration. If it is more than 250-μm deep, the risk of endothelial cell loss related to UV-A riboflavin is higher. Furthermore, the effectiveness of this treatment is lower the deeper the infiltrate. Fungal infections penetrate deeper than bacterial and this pattern can explain why the treatment is less effective and more dangerous. In the study of Müller [37], a patient infected with *Candida* and treated by CXL developed a corneal edema due to endothelial failure. Panda [35] described nine mechanisms of action in the healing of corneal ulcers such as (1) inactivation of pathogens by direct damage to bacterial DNA [40], (2) increased resistance to enzymatic degradation [21] initiated by the increase in covalent bonds in the corneal stroma causing regression in ulcer morphology, (3) increased stromal tensile strength and rigidity of corneal collagen, which may prevent melting [41], (4) wound healing by induction of apoptosis followed by repopulation restoring the normal cytoarchitecture of the cornea [15], (5) reduced susceptibility of the tissue to the organism due to ultrastructural change [42], (6) corneal epithelial surface reformation due to the ability of riboflavin in the development and maintenance of the surface structures of the epithelial cells [43], (7) chemical alteration of functional groups of nucleic acids in the bacteria, making replication impossible [44], (8) reduction in inflammatory and immune cells [44], (9) reduced nociceptive response of corneal nerves that decrease pain [44] and reduced tendency to generate vascularization [43]. Another advantage of CXL is to avoid emergency keratoplasty. This minimizes reinfection and should provide the opportunity to perform lamellar grafts. It would also be useful to reduce the risk of rejection. The rejection rate after emergency keratoplasty is much higher than standard keratoplasty [45]. There are only a small number of studies available due to the ethical problem of selecting patients to treat with CXL without antibiotic therapy affecting the result. In the majority of these studies, this treatment was chosen for recurrent and unresponsive keratitis. Only the patients from the study of Makdoumi [24] were not at a severe stage. This is the only study in which the patient did not have antibiotic drops.

## Conclusion

In conclusion, our observations show that corneal CXL could be a new interesting way of handling infectious keratitis. CXL treatment is supported by evidence of effectivity in the control of infectious keratitis stopping the progress of corneal melting, but the absence of control groups in the studies published to date does not allow us to indicate this treatment as immediately feasible. The treatment seems to be more effective in blocking corneal melting in bacteria and *Acanthamoeba* than in fungus, but the different grades of severity of keratitis and the absence of standardization to evaluate this do not allow us to provide a clear final suggestion. Furthermore, a recent article [46] showed a contradictory outcome in the application of CXL in rabbits infected with Acanthamoeba keratitis, stating that this treatment was not effective. On the other hand, another author [47] proposed a combined treatment with amphotericin B and CXL in fungal infection. In this way, the drug interacts with the fungal membrane sterols forming transmembrane channels and allows the riboflavin to enter into the cell and then destroy its activity. We have to bear in mind also that CXL with UV-A and riboflavin reduces corneal permeability [48] and how this issue can affect postoperative treatment. The expected complications of CXL in infectious keratitis are endothelial cell loss related to fungal deep infiltration and reactivation of previous herpes simplex. With the intention of avoiding these complications, we propose the following: first, to exclude all patients with a previous history of herpes keratitis, second, to use hyposmolarity riboflavin when a deep infiltration is observed. At present, CXL should be considered in cases of severe unresponsive infectious keratitis before undertaking emergency keratoplasty. A study to compare CXL with standard topical antibiotic treatment in which the severity of keratitis and the infecting organism are homogeneous is required to clarify and prove its application.

## Abbreviations

CXL: Collagen cross linking; UV-A: Ultraviolet-A; BCVA: Best-corrected visual acuity.

## Competing interests

The authors declare that they have no competing interests.

## Authors’ contributions

AA collected and analyzed the data, wrote the article, and performed the statistical analysis. JLA designed the study, wrote the article, and performed critical revision of the manuscript. JAG designed the study and performed a critical revision. DDV and JMBC collected the data and performed a critical revision. All authors read and approved the final manuscript.
